# Performance of Ground-Based Solar-Induced Chlorophyll Fluorescence Retrieval Algorithms at the Water Vapor Absorption Band

**DOI:** 10.3390/s25030689

**Published:** 2025-01-24

**Authors:** Yongqi Zhang, Xinjie Liu, Shanshan Du, Mengjia Qi, Xia Jing, Liangyun Liu

**Affiliations:** 1 College of Geomatics, Xi’an University of Science and Technology, Xi’an 710054, China; 2 International Research Center of Big Data for Sustainable Development Goals, Beijing 100094, China; 3 Key Laboratory of Digital Earth Science, Aerospace Information Research Institute, Chinese Academy of Sciences, Beijing 100094, China

**Keywords:** solar-induced chlorophyll fluorescence (SIF), water vapor absorption band, tower-based observations, SIF algorithm comparison, error analysis, sensitivity analysis

## Abstract

Solar-induced chlorophyll fluorescence (SIF) is essential for monitoring vegetation photosynthesis. The water vapor absorption band, the broadest absorption window, has a deeper absorption line than the O_2_-B band, providing significant potential for SIF retrieval; however, substantial variation in atmospheric water vapor column concentrations limits research on SIF retrieval using this band. This study evaluates seven common SIF retrieval algorithms, including sFLD, 3FLD, iFLD, pFLD, SFM, SVD, and DOAS, using simulated datasets under varying atmospheric water vapor concentrations, spectral resolution (SR), and signal-to-noise ratios (SNRs). Additionally, the SIF retrieval results from the H_2_O, O_2_-B, and O_2_-A absorption bands are compared and analyzed to explore the fluorescence retrieval potential of the water vapor band. Furthermore, the potential of commonly used spectrometers, including Ocean Optics QE Pro and ASD FieldSpec 3, for SIF retrieval using the water vapor absorption band was evaluated. The results were further validated using ground-based tower observations. The results show that sFLD consistently overestimates SIF in the water vapor band, limiting its reliability, while SFM performs best across varying conditions. In comparison, 3FLD and pFLD, along with SVD, are accurate at high resolutions but less effective at lower ones. iFLD performs relatively poorly overall, whereas DOAS excels in low SR retrievals. At the same time, our study also shows that the water vapor band offers higher accuracy in ground-based SIF retrieval compared to the O_2_-B band, demonstrating strong application potential and providing valuable references for selecting SIF retrieval algorithms.

## 1. Introduction

Solar-induced chlorophyll fluorescence (SIF) serves as a key indicator of photosynthetic activity across diverse observational scales [1]; however, the weak emission of SIF signals and their spectral overlap with solar radiation reflected by vegetation present inherent challenges for direct measurement [2]. With advancements in remote sensing technology, utilizing atmospheric absorption and Fraunhofer line infilling effects has made SIF retrieval possible [3,4].

Traditionally, SIF data acquisition has primarily relied on satellite platforms [5,6], playing a key role in large-scale vegetation photosynthesis monitoring; however, with an increase in the number of flux towers equipped with SIF-monitoring spectrometers, ground-based platforms can now provide new opportunities for localized high-resolution observations, particularly for monitoring dynamic SIF fluctuations and diurnal variations related to environmental drivers such as moisture, temperature, and atmospheric conditions [7,8,9,10]. Despite these advances, SIF retrieval from tower-based platforms still confronts challenges, particularly due to atmospheric radiative transfer effects and systematic and random errors within the retrieval algorithms themselves [11,12,13].

**Figure 1:**
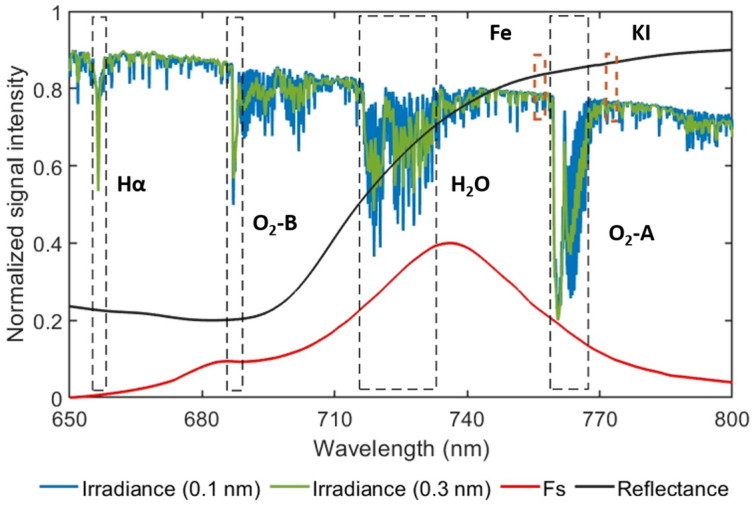
Irradiance with SRs of 0.1 nm and 0.3 nm (with spectral sampling intervals of 0.05 nm and 0.15 nm, respectively), simulated chlorophyll fluorescence (Fs), and reflectance spectra. (The dashed box indicates the range of each absorption band).

Challenges in SIF retrieval are related to the selection of absorption bands, with each presenting unique characteristics and retrieval complexities. Figure 1 illustrates the absorption bands commonly used for SIF retrieval. The O_2_-B band, situated in the red spectral region, is highly sensitive to dynamic variations in vegetation reflectance spectra, which are influenced by biophysical and biochemical parameters [14]. These variations exacerbate biases in reflectance assumptions, leading to marked declines in retrieval accuracy. The O_2_-A band, recognized for its deep absorption lines and broad window, provides significant potential for SIF retrieval; however, it is strongly affected by atmospheric radiative transfer, complicating the corrections required when using tower-based data [15]. In contrast, Fraunhofer lines, such as Fe and KI lines, require much higher spectral resolutions (SRs) to achieve reliable retrieval results [16,17]. The H_α_ band is a relatively wide Fraunhofer band, but the SIF signal at this band is too weak for precise retrieval.

Considering the limitations of these commonly used bands, the 719 nm water vapor band also has potential for SIF retrieval but has rarely been used. This band exhibits deeper absorption features than the O_2_-B band and aligns closely with the SIF spectral peak, suggesting enhanced sensitivity and accuracy in SIF retrieval. Additionally, the absorption window of the 719 nm water vapor band is broader compared with the O_2_-B and O_2_-A bands, and this wider window improves SIF retrieval by capturing a broader spectral range, enabling more robust and accurate fluorescence signal extraction. Moreover, Liu noted that the depth of absorption lines in the downwelling spectrum directly correlates with retrieval accuracy [18], further emphasizing the potential of the water vapor band. On the other hand, the H_2_O band, located between the two fluorescence peaks, is critical for capturing the overall shape of chlorophyll fluorescence spectra.

Despite these benefits, the water vapor band has its limitations. First, variations in atmospheric water vapor concentrations during observations introduce stability issues, which can lead to inconsistencies in SIF data across temporal and spatial scales [19]. Second, the water vapor band is located at the “red-edge” region of the vegetation reflectance spectra, leading to difficulties in accurate modeling of the reflectance spectral shape, which is important for SIF retrieval.

The QE65 Pro spectrometer (Ocean Optics, Dunedin, FL, USA), a commonly used spectral data acquisition instrument, is widely applied in research on the O_2_ absorption bands due to its prominent advantage of high resolution. For fluorescence retrieval studies in the O_2_-A and O_2_-B bands, a resolution of 1 nm or higher is typically required to ensure sufficient retrieval accuracy, making the QE65 Pro instrument irreplaceable. This also holds true for our research on the water vapor absorption band. Additionally, the ASD FieldSpec 3 spectrometer (Analytical Spectral Devices, Boulder, CO, USA) is commonly used in field reflectance observations and other experimental studies. Its resolution is 3 nm, which is lower compared to the QE65 Pro, but considering the wider absorption window of the water vapor band, we simulated the payload configuration of this instrument in our subsequent research. The accuracy and reliability of the results will directly support the capability of this instrument for fluorescence retrieval in the water vapor absorption band. Through this, we aim to deepen our understanding of SIF retrieval, particularly in the water vapor absorption band, and provide valuable insights into the fluorescence retrieval potential of this spectral region. This study aims to investigate the potential of SIF retrieval from water vapor channels using ground-based data, focusing on three key objectives: (1) evaluating the applicability of different SIF retrieval algorithms in water vapor absorption bands; (2) analyzing the reliability of SIF data obtained from various algorithms under different instrument configurations; and (3) exploring the robustness and accuracy of SIF retrieval algorithms in the presence of significant variations in atmospheric water vapor concentrations.

## 2. Materials and Methods

Considering that the true values of SIF cannot be obtained in actual observations, we used simulated data for the related experiments. However, simulated experiments have their limitations as they cannot fully replace all field observation environments. Therefore, we subsequently added some tower-based observational experiments to supplement the study.

### 2.1. Simulated Dataset

In this study, 324 sets of simulated fluorescence and reflectance data were generated using the soil–canopy–observation of photosynthesis and energy balance (SCOPE) model, while atmospheric parameters were simulated with the moderate-resolution atmospheric transmission (MODTRAN) model. Both models’ input values were based on parameters from the EU LOPEX’93 experimental dataset, as detailed in Table 1, with all other parameters set to their default values.

To integrate atmospheric data generated by MODTRAN with the reflectance spectra and SIF signals from the SCOPE model, the following radiative transfer equation was referenced [20]:

(1) $$L_{Sensor}=\frac{E}{\pi }\times R+SIF$$

where $L_{Sensor}$ represents the upward radiance signal at the sensor height, E is the downwelling irradiance, R is the reflectance, and SIF is the fluorescence contribution. Additionally, Gaussian random noise was introduced to examine the effects of varying signal-to-noise ratios (SNRs), specifically set at 300, 500, 1000, and 3000. To evaluate the performance of the algorithms under the technical specifications commonly experienced in field experiments by spectrometers, this study simulated the corresponding SR and SNR values. The QE65 Pro has an SR of 0.31 nm and an SNR of 1000, while the ASD FieldSpec 3 spectrometer has an SR of 3 nm and an SNR of 4000.

### 2.2. Field Measurements

**Figure 2:**
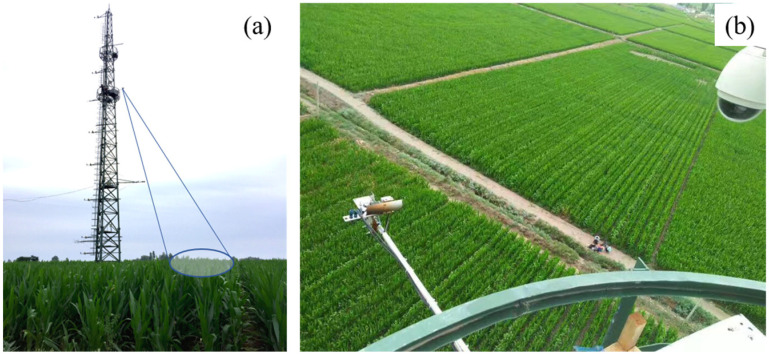
ChinaSpec Daman Oasis Farmland Station (Observation height: 25 m; underlying surface: maize fields). (**a**) Aerial view of the DM Observation Station; (**b**) Observation equipment on the tower.

Ground-based observational experiments were conducted at Daman (DM) Station (Figure 2), located in the agricultural oasis of Zhangye, Gansu Province, China. This site, characterized by its favorable geographic location and typical farmland ecological features, represents an ideal location for studying the spectral characteristics of agricultural ecosystems. The station’s underlying surface is predominantly used to cultivate maize, and the experiments spanned the entire maize growing season (June to September) in 2021. Detailed canopy spectral data were collected across various growth stages, providing a high-quality observational basis for fluorescence retrieval studies. The ChinaSpec spectral observation system used in this study is equipped with a high-performance QE65 Pro spectrometer, fitted with a cosine corrector and a bare fiber, with a field of view of 25°, a zenith angle of 25°, and an azimuth angle of 210°. We installed the instrument on an eddy covariance (EC) tower 25 m above the ground for experimental observations. The instrument housing is equipped with a beam splitter connected to two optical fibers, which are used to measure downwelling irradiance and upwelling radiance data, respectively. Additionally, the instrument is fitted with a cosine corrector for acquiring downwelling irradiance, while a bare fiber is used for measuring upwelling radiance. The spectrometer has a spectral resolution (SR) of 0.31 nm and a sampling interval of 0.155 nm, covering a wavelength range from 650 nm to 815 nm, ensuring precise fluorescence signal measurements. By capturing both the upwelling and downwelling radiation spectra, the system can effectively characterize a canopy’s spectral features, ensuring high data quality while supporting the validation and refinement of fluorescence retrieval algorithms.

A “sandwich-style” observation approach was employed during these measurements, involving alternating acquisitions of downwelling irradiance and upwelling radiance. This design significantly reduced the errors associated with temporal mismatches between these measurements, enhancing the reliability and accuracy of experimental data. To ensure data quality, the raw spectral data obtained were subsequently manually inspected, and quality screening was performed to exclude spectral outliers caused by instrument malfunctions or other anomalies. The resulting dataset provides critical support for subsequent tower-based experimental validations.

### 2.3. Validation Method

Existing studies have shown that for ground-based spectral observation data with a spectral resolution of 0.3 nm, the 3FLD algorithm exhibits strong robustness and simplicity in computation, making it a commonly used algorithm for ground-based SIF retrieval [18]. In our experiment, since true SIF values cannot be directly obtained from field measurements, the retrieval results of the 3FLD algorithm in the O_2_-A band were used as a reference. Note that, due to the strong atmospheric radiative transfer absorption effects in the O_2_ absorption bands, atmospheric correction was carried out before SIF retrieval. To ensure comparability with the results of the 3FLD algorithm in the O_2_-A band, a linear relationship was assumed to exist between the chlorophyll fluorescence spectra in the two spectral bands. The relationship between the 760.6 nm (O_2_-A) and 718.9 nm (H_2_O) bands was calculated using the simulated fluorescence spectra. Ultimately, a conversion ratio of 1.2626 was determined based on the slope, which was used to convert the SIF retrieval values from the O_2_-A band to the H_2_O band [21].

In addition, in the simulation and tower-based observation experiments, we used root mean square error (*RMSE*) and relative root mean square error (*RRMSE*) as accuracy evaluation metrics, with the corresponding calculation formulas as follows:

(2) $$RMSE=\sqrt{\;\frac{1}{n}\sum_{i=1}^{n}{\left(y_{i}-{\hat{y}}_{i}\right)}^{2}}$$

(3) $$RRMSE=\frac{RMSE}{\bar{y}}\times 100$$

where $\;y_{i}\;$ is the observed (true) values, $\;{\hat{y}}_{i}\;$ is the predicted (estimated) values, n is the number of data points, and $\;\bar{y\;}$ is the mean of the observed (true) values.

### 2.4. SIF Retrieval Algorithms

SIF retrieval capitalizes on Fraunhofer dark lines and atmospheric absorption features that establish spectral windows to amplify often-overlooked SIF signals. Several algorithms have been developed based on the principle of Fraunhofer line filling, including the standard Fraunhofer line discrimination (FLD) algorithm, the improved three-band FLD (3FLD), the improved FLD (iFLD), and the principal component analysis (PCA)-based FLD (pFLD). Additional noteworthy approaches comprise the spectral fitting method (SFM), singular value decomposition (SVD), and differential optical absorption spectroscopy (DOAS) (as illustrated in Table 2). This study utilized the aforementioned algorithms for SIF retrieval in the water vapor band, targeting individual spectra and conducting experiments under varying weather conditions. Initially, SIF data were obtained at 3-min intervals, and subsequently, we averaged the 3-min SIF data to 30-min intervals for use in our experiments. It is important to note that the forward model of the SFM algorithm used in this study is presented in the table below. In subsequent research, we demonstrated that the absorption effect in the water vapor band is weak, thus allowing the neglect of atmospheric correction. This forward model was also applied to the O_2_ absorption band. Furthermore, for the SVD algorithm, all downwelling radiance measurements from each day were utilized for training [22].

Additionally, different retrieval algorithms require adjustments to their retrieval windows based on variations in SRs. Through multiple simulation experiments and subsequent analysis, we optimized the retrieval windows, with the selected windows summarized in Table 3. It is important to note that the SVD algorithm relies on a substantial amount of spectral information due to its underlying principles, necessitating a broader retrieval window at a 3.0 nm SR to obtain valid SIF data. However, the reliability of the results for the water vapor absorption band at this resolution may be compromised. Consequently, the results of this algorithm at a 3.0 nm SR in this study should be regarded as preliminary and for reference only.

## 3. Results

### 3.1. Assessing SIF Retrieval Accuracy with SCOPE and MODTRAN Simulations

#### 3.1.1. Influence of SR and SNR on SIF Retrieval Accuracy

**Figure 3:**
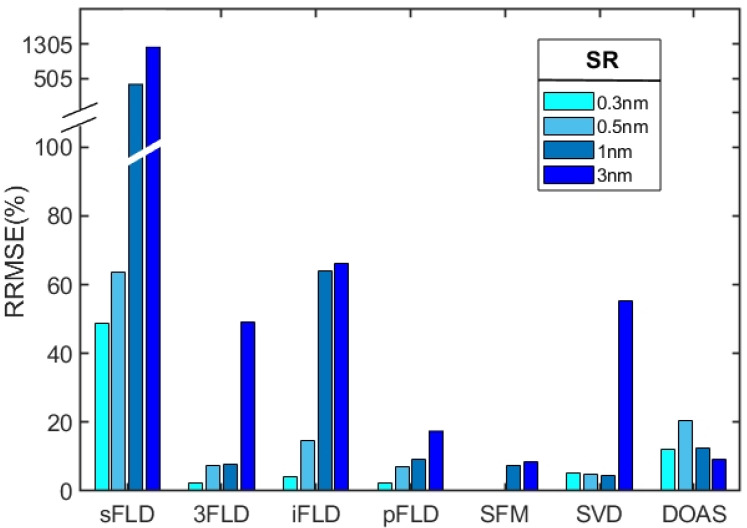
Comparison of retrieved SIF values across the seven algorithms at varying SRs, with the water vapor concentration held at a midlevel value of 3.0 g/cm^2^ and no added noise. The color gradient reflects different resolution levels, while “discontinuity” symbols denote omitted intermediate values because of excessively high numerical errors.

To assess the retrieval accuracy of the algorithms at different SRs, the atmospheric parameters were set to the default values, fixing the water vapor column concentration at a midrange level of 3 g/cm^2^. Under conditions devoid of random noise influences, data with various SR levels were utilized to compare the performance of the seven SIF retrieval algorithms. The RRMSE was calculated for each algorithm (Figure 3). The performance analysis reveals that the sFLD algorithm demonstrates the least effectiveness, exhibiting RRMSE values exceeding 100% at lower SRs. In contrast, the SFM algorithm consistently achieves superior accuracy, maintaining low retrieval errors across all tested SRs. Both 3FLD and pFLD exhibit strong performances at high SRs; however, their retrieval errors increase significantly at coarser resolutions. The iFLD algorithm, while performing well at high resolutions, experiences a notable decline in precision at 1 nm and 3 nm. The SVD algorithm maintains stable accuracy at higher SRs, but its effectiveness diminishes at coarser resolutions, likely due to the loss of high-frequency information. Additionally, at a 3 nm resolution, an extremely broad retrieval window is required to obtain SIF results, compromising the representativeness of the water vapor band; therefore, the SVD retrieval results under a 3 nm resolution are provided for reference only. Overall, SFM emerges as the most reliable algorithm, particularly at elevated SRs.

**Figure 4:**
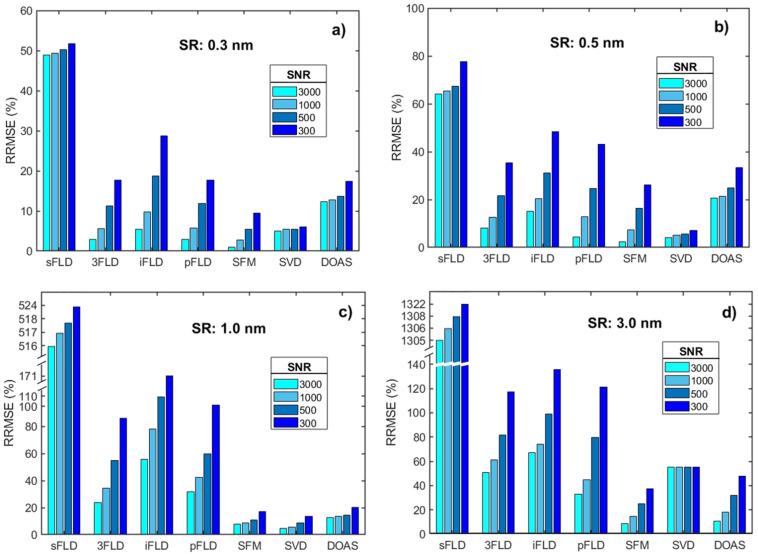
RRMSE values of SIF retrieved by the seven algorithms in the 719 nm water vapor band under different SNR conditions. The SRs used to generate these data are 0.3 nm (**a**), 0.5 nm (**b**), 1.0 nm (**c**), and 3.0 nm (**d**), with a constant water vapor column concentration of 3 g/cm^2^.

In addition to the sensitivity analysis of SRs, the impact of noise was also considered (Figure 4). At a high SR of 0.3 nm, the sFLD algorithm demonstrates significant retrieval errors, with RRMSE values consistently around 50% across varying SNRs. This poor performance is attributable to intrinsic retrieval errors in the water vapor band. In contrast, the 3FLD, iFLD, pFLD, and SFM algorithms show increased errors as noise levels increase, with SFM exhibiting the best overall performance. The SVD algorithm remains stable across different noise conditions, maintaining an RRMSE value of approximately 6%; this may be attributed to its data-driven approach, which results in strong robustness against external noise interference. With a reduction in SR, retrieval errors increase noticeably across all algorithms. At a resolution of 0.5 nm, although errors for each algorithm grow significantly, they still provide generally reliable retrieval information; however, when the SR reaches 1 nm, sFLD loses its reliability entirely, and the other three FLD algorithms perform poorly under high noise conditions. Notably, SFM, SVD, and DOAS continue to show strong performance. At an SR of 3 nm, the retrieval error for sFLD increases further, while the remaining algorithms (3FLD, iFLD, pFLD, SFM, and DOAS) display consistent trends, with SFM achieving the highest accuracy (RRMSE of 42.5% at an SNR of 300), followed by DOAS. Notably, the SVD algorithm, with an RRMSE value of approximately 60%, performs better than the four FLD algorithms overall; however, as discussed previously, the limited persuasiveness of the retrieval window at 3 nm suggests that SVD may be less suitable at this resolution.

#### 3.1.2. The SIF Retrieval Accuracy Under the Payload Configurations of the QE65 Pro and ASD FieldSpec 3 Spectrometers

In order to investigate the applicability of two commonly used spectrometers (QE65 Pro and ASD FieldSpec 3) for SIF retrieval in the water vapor absorption band, we simulated the corresponding technical specifications for both instruments, as described in the methodology section above. The SIF retrieval results for each algorithm under these technical specifications are presented in Table 4.

Under the specifications of the QE Pro spectrometer, the sFLD algorithm shows the highest retrieval error, with an RRMSE value of 49.4%. Notably, except for the sFLD algorithm, the remaining six methods demonstrate comparable performance. When evaluated under the ASD specifications, the sFLD algorithm becomes entirely unreliable, while the 3FLD, iFLD, and SVD algorithms experience increased retrieval errors, exceeding RRMSE values of 50%. Although pFLD shows slightly superior performance, its error still reaches 31.75%. Notably, the SFM and DOAS algorithms achieve the lowest errors at 9.08% and 9.56%, respectively.

#### 3.1.3. Sensitivity of SIF Retrieval Algorithms to Water Vapor Concentration

**Figure 5:**
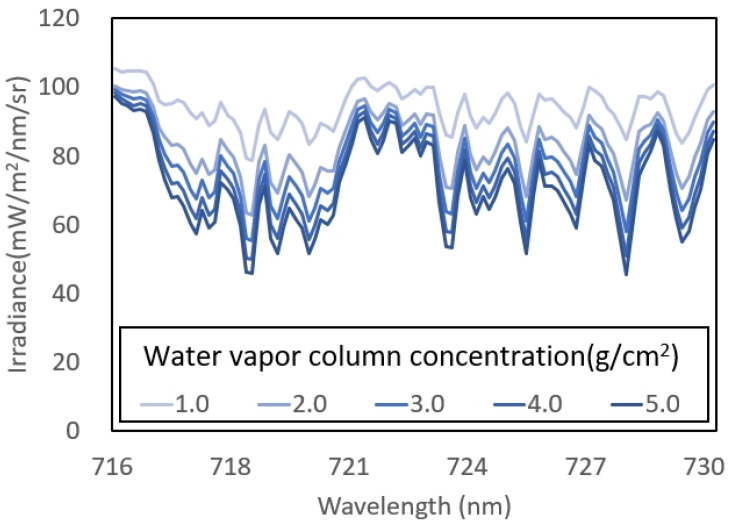
Simulated downwelling irradiance in the water vapor band (716–730 nm) at varying water vapor column concentrations, with the SR set to 0.3 nm, excluding other atmospheric parameters.

In this study, how the variation in atmospheric water vapor concentration affects the retrieval of SIF was explored by evaluating the performance of seven algorithms: sFLD, 3FLD, iFLD, pFLD, SFM, SVD, and DOAS. To simplify the analysis and ensure that the variations in SIF retrieval accuracy primarily arise from changes in water vapor column concentrations, the instrument configuration was fixed at an SR of 0.3 nm and excluded Gaussian random noise. The focus of this study was the impact of changes in water vapor column concentration on SIF retrieval accuracy. Thus, the atmospheric water vapor column concentration was varied within the range of 1.0 g/cm^2^ to 5.0 g/cm^2^. The findings reveal intriguing insights into the sensitivity of these algorithms to changes in water vapor concentration. Previous studies have demonstrated that the depth of absorption lines directly impacts the accuracy of SIF retrieval. As the atmospheric water vapor concentration increases, these data clearly show a reduction in downwelling irradiance within the water vapor absorption bands, accompanied by a deepening of the absorption lines (Figure 5). This pattern indicates that a higher concentration of atmospheric water vapor enhances the accuracy of SIF data, further supporting the conclusion that the depth of water vapor absorption lines plays a critical role in the performance of SIF retrieval algorithms.

**Figure 6:**
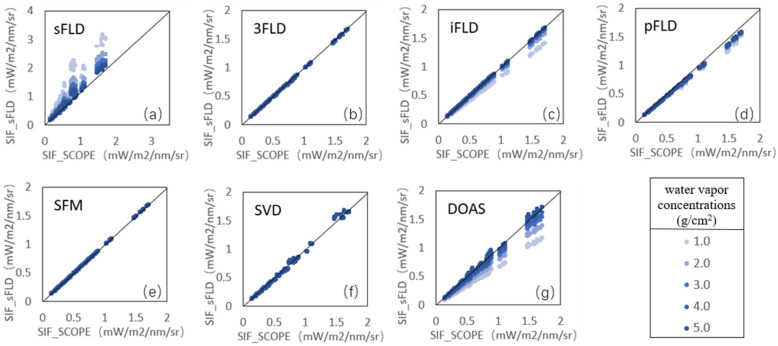
SIF retrieval across water vapor concentrations (1.0 g/cm^2^ to 5.0 g/cm^2^) with an SR of 0.3 nm in the absence of Gaussian noise, with scatter point colors representing water vapor concentrations from low (1.0 g/cm^2^) to high (5.0 g/cm^2^) in increasing intensity (Where (**a**–**g**) represents the results of the seven different retrieval algorithms).

Notably, the scatter plot in Figure 6 visually depicts the relationship between SIF retrieval results and water vapor concentrations, with points exhibiting a gradient from light to dark as water vapor levels increase. Among the algorithms, sFLD, iFLD, and DOAS showed heightened sensitivity to fluctuations in water vapor, resulting in notable discrepancies in their SIF estimates. Conversely, algorithms such as 3FLD, SFM, and SVD demonstrated more robust performance, maintaining a close alignment with the 1:1 reference line. Additionally, while sFLD consistently overestimated the SIF values, pFLD, iFLD, and DOAS tended to underestimate them, a trend likely contributing to their higher error rates compared with the other methods. This can be attributed to the complex absorption features caused by atmospheric water vapor absorption, which imposes limitations on each algorithm when handling intricate spectral characteristics.

**Figure 7:**
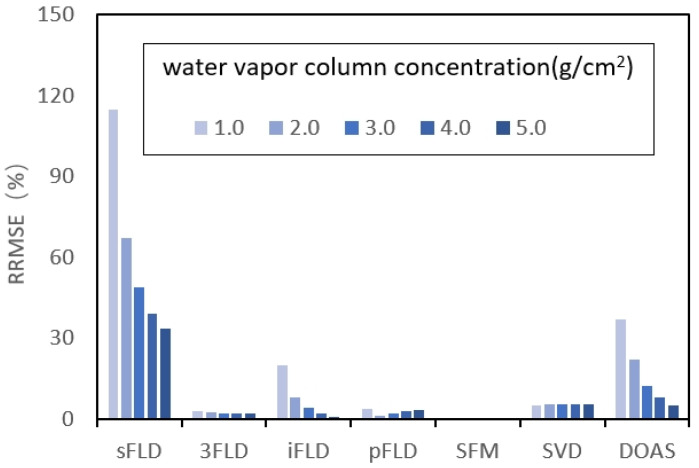
RRMSE values of retrieved SIF from the seven algorithms under varying water vapor column concentrations, utilizing different SRs in the absence of Gaussian random noise.

The sensitivity of the algorithms to variations in atmospheric water vapor concentrations is illustrated in Figure 7. The heights of the bars for 3FLD, pFLD, SFM, and SVD remain consistent across different water vapor column concentrations, indicating their robust performance. In contrast, sFLD, iFLD, and DOAS exhibit a trend of decreasing errors with increasing water vapor concentration, demonstrating their sensitivity to atmospheric conditions. Notably, the RRMSE value of SFM approaches zero, resulting in an almost invisible bar in the histogram, underscoring its effectiveness in SIF retrieval. It indicates that the spectral fitting method effectively handles complex spectral characteristics, especially in the complex environment of the water vapor band, allowing for efficient differentiation between signal and noise.

Considering the impact of atmospheric water vapor column concentrations, the algorithms sFLD, iFLD, and DOAS exhibit heightened sensitivity to water vapor fluctuations. In contrast, 3FLD, pFLD, SFM, and SVD demonstrate superior robustness, with the SFM method notably achieving the lowest retrieval errors. This disparity in performance underscores the importance of selecting appropriate algorithms for SIF retrieval in varying atmospheric conditions, as the stability provided by the latter group enhances accuracy and reliability. Overall, these findings contribute to a deeper understanding of how atmospheric water vapor influences the efficacy of SIF retrieval methodologies.

#### 3.1.4. Comparison and Analysis of Multi-Band SIF Retrieval Results

To highlight the potential of fluorescence retrieval in the water vapor absorption band, this study incorporates SIF retrieval results from both the O_2_-B and O_2_-A bands, allowing for a comparative analysis of the three spectral bands. Specifically, the analysis was conducted under a water vapor column concentration of 3 g/cm^2^, with spectral resolutions (SR) of 0.3 nm and 3.0 nm, and without the addition of Gaussian random noise. The accuracy of the retrieval results was evaluated using three performance metrics: correlation coefficient (R^2^), relative root mean square error (RRMSE), and slope (the slope of the linear regression); the results are shown in Table 5.

Under a spectral resolution of 0.3 nm, the R^2^ values for all algorithms across the three retrieval windows remained close to 1, indicating a high goodness of fit for the SIF retrievals in all bands. From the perspective of RRMSE, the SIF retrieval accuracy generally followed the trend O_2_-A > H_2_O > O_2_-B. Notably, the sFLD algorithm exhibited anomalous behavior, with higher errors in the H_2_O band compared to the O_2_-B band. This is likely linked to the severe overestimation previously identified in sFLD, which is further reflected in the slope analysis, where the slope deviated significantly from 1 in the H_2_O band. In contrast, the slopes of other algorithms remained close to 1.0, with no significant differences observed.

At a spectral resolution of 3.0 nm, the retrieval accuracy in the O_2_-A band was considerably higher than in the O_2_-B and H_2_O bands across all accuracy metrics. Specifically, the SIF retrievals in the O_2_-A band were superior to those in the other bands, as indicated by the R^2^, RRMSE, and slope values. In the H_2_O band, although the R^2^ values were superior to those in the O_2_-B band, the RRMSE values for sFLD and iFLD were notably higher. This can be attributed to severe overestimation in the case of sFLD and potential atmospheric water vapor absorption effects for iFLD. For pFLD, the retrieval accuracy in the H_2_O band was slightly lower than that in the O_2_-B band, although the difference was not statistically significant.

In conclusion, these results further confirm the effectiveness of the H_2_O absorption band for SIF retrieval, with most algorithms yielding better accuracy in the H_2_O band compared to the O_2_-B band. Nevertheless, the O_2_-A band remains the optimal choice for SIF retrieval, exhibiting the best fluorescence retrieval performance.

### 3.2. Assessing SIF Retrieval Accuracy with Field Measurements

#### 3.2.1. Comparison with SIF Retrieved at the O_2_-A Band

Although various instrument configurations and atmospheric conditions have been simulated, these simulations cannot fully represent an entire experimental environment; therefore, supplementing the results with ground-based tower data is performed. The relevant experimental validation methods have been described in the previous section on experimental methods and are not reiterated here.

**Figure 8:**
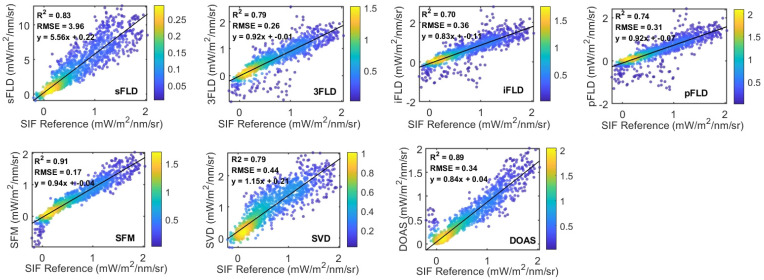
R^2^ and RMSE calculations comparing SIF retrieved from the water vapor band using ground observation data from the DM station in 2021 with SIF obtained from 3FLD in the O_2_-A band. The color bar on the right of the density plot represents the range of density values, with scatter points colored from blue to yellow, indicating increasing density from low to high.

To rigorously evaluate the retrieval accuracy, this study used 30-min averaged SIF data to calculate the RMSE and R^2^ between the fluorescence retrieval values in the water vapor band and the reference values derived from the O_2_-A band using the 3FLD algorithm (Figure 8). Ground-based spectral data were collected at the DM station during the growing season from June to September 2021. The results indicate that although the retrieval errors of the sFLD algorithm are notably higher than those of other methods, its higher R^2^ values suggest strong data consistency with the SIF reference values. Excluding sFLD, all algorithms exhibited RMSE values below 1.0. The SFM algorithm demonstrated the highest retrieval accuracy. In contrast, iFLD showed relatively lower accuracy, while the differences in accuracy between the other algorithms were not significant. This is generally consistent with the conclusions drawn from the simulation experiments, though some differences were observed, which we attribute to the limitations of the simulations in fully replicating the actual observational environment.

Additionally, considering that weather conditions (such as sunny and cloudy days) may affect the accuracy of SIF (solar-induced chlorophyll fluorescence) retrieval, we classified the data into sunny and cloudy conditions. A value of 930 mW/m^2^/nm for the downward radiation irradiance at 780 nm divided by cos (SZA) was used as a threshold to classify the weather conditions. We then calculated the R^2^ and RMSE for each part using the method described earlier, and the results are shown in Table 6.

The results indicate that most algorithms exhibit higher retrieval accuracy under sunny conditions compared to cloudy conditions. This advantage is not significant in terms of RMSE, but it is more apparent in R^2^. Notably, the sFLD algorithm shows abnormal behavior, which we suspect is due to severe overestimation of SIF, and the influence of sunlight may have counteracted this overestimation, leading to a reduction in RMSE.

**Figure 9:**
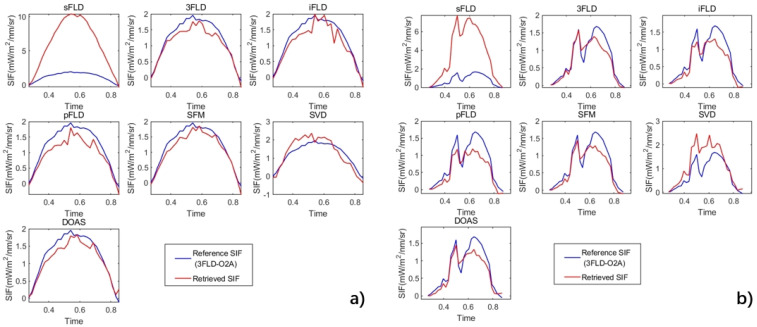
Solar-induced chlorophyll fluorescence (SIF) was retrieved from the water vapor band using seven algorithms, based on observations from the DM station in China. (**a**) Results for 7 July 2021 (sunny), and (**b**) results for 11 July 2021 (cloudy). The SIF data are transformed through ratio calculations and compared with the normalized SIF obtained from the 3FLD algorithm in the O_2_-A band, using 30-min averaged SIF data.

Furthermore, to analyze the overestimation and underestimation behaviors of fluorescence under different weather conditions, fluorescence retrieval data from the DM station on 7 July 2021 (sunny day) and 11 July 2021 (cloudy day) were extracted. Daily variation curves were plotted based on the band conversion method described earlier, with the results shown in Figure 9. The blue curve illustrates the daily variation in solar-induced chlorophyll fluorescence retrieved using the 3FLD algorithm in the O_2_-A band, while the red curves represent the results from various algorithms in the water vapor band. The sFLD algorithm significantly overestimates SIF, resulting in high retrieval errors, whereas the 3FLD, iFLD, pFLD, SFM, and DOAS algorithms show varying degrees of underestimation. This trend is consistent under both weather conditions. Overall, compared to the results obtained from the simulation experiments, slight discrepancies were observed in the actual observational data, which may arise from the inherent limitations of simulations in accurately reflecting real-world conditions.

#### 3.2.2. Seasonal Patterns of SIF Retrieved by Different Algorithms

**Figure 10:**
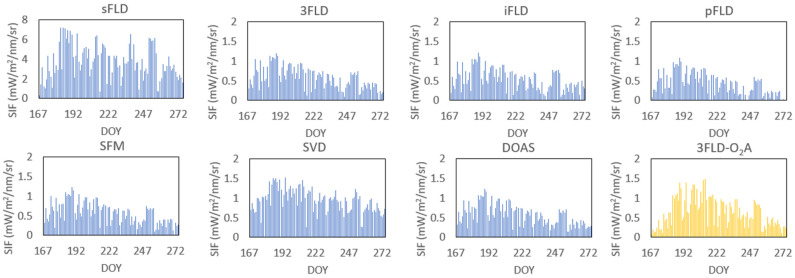
Comparison of the daily mean SIF variation derived using seven algorithms in the water vapor band from spectral data collected at the DM station in China during the entire maize phenological stage from June to September 2021, with the results of the 3FLD algorithm in the O_2_-A band. To facilitate differentiation, the results of the 3FLD algorithm in the O_2_-A band are color-coded in yellow. Similarly, using the 3FLD retrieval values in the O_2_-A band as reference values, the results show that, overall, the retrieval values of all algorithms in the water vapor band exhibit good consistency with the 3FLD retrieval values in the O_2_-A band throughout the entire phenological stage. Both show the overall trend of SIF values increasing first and then decreasing with the increase in DOY, reaching the highest point in late July. This indicates that, although different retrieval algorithms have different processing methods and assumptions, they show certain similarities in capturing the temporal dynamics of the crop growth cycle. At the same time, the consistency between the SIF retrieval results in the water vapor band and the O_2_-A band also suggests that this band plays a crucial role in capturing the dynamic changes in plant growth and photosynthesis, particularly during different phenological stages of crop growth.

Solar-induced chlorophyll fluorescence (SIF) is closely related to the phenological stages of crops. Therefore, we consider analyzing the data along the DOY (Day of Year) timeline, aiming to assess the potential impact of retrieval algorithms in capturing the temporal dynamics between SIF and the crop growth cycle. We used spectral data from the DM station collected between June and September 2021 for the corresponding fluorescence retrieval and calculated the daily average SIF values. The results are shown in Figure 10.

## 4. Discussion

This study explores the potential of the water vapor absorption band for ground-based SIF retrieval, highlighting its advantages and inherent limitations. Meanwhile, based on the simulated payload configurations of two commonly used spectrometers, the QE65 Pro and the ASD FieldSpec 3, we demonstrated the applicability of these instruments in the water vapor absorption band. The former ensures sufficient spectral resolution and signal-to-noise ratio under complex atmospheric water vapor absorption conditions [30], while the latter, under low-resolution observation conditions [31], highlights the advantages of the broad band range in the water vapor absorption region, providing new insights for fluorescence retrieval in this band.

Positioned in the red-edge spectral region, spectral reflectance is relatively lower compared with the commonly used O_2_-A absorption band, which enhances the separation of SIF from background noise and improves retrieval accuracy [4,32]. Simulations and ground-based tower experiments confirm that fluorescence signals within this band remain robust and accurate, even under high atmospheric water vapor concentrations; however, the band’s location within the “red-edge” spectral region introduces a complex reflectance spectrum [25]. Variations in atmospheric water vapor concentration significantly influence spectral profiles, complicating spectral fitting and reducing the stability of retrieval results.

The relatively broad width of the water vapor absorption band provides advantages for fluorescence retrieval, particularly by providing additional sampling points that enhance the performance of spectral fitting algorithms [33]. Algorithms such as SFM and DOAS demonstrate reliable retrieval performance and high accuracy even at lower SRs, benefiting significantly from this broader band. Similarly, the SVD algorithm, which relies on a higher number of sampling points, achieves excellent retrieval accuracy at a resolution of 1 nm. These findings highlight the advantages conferred by a broader retrieval window, supporting fluorescence retrieval and enhancing algorithm adaptability in this spectral region. Given the spectral characteristic nature of the ASD FieldSpec 3 spectrometer, simulating its configuration for retrieval studies in this band is highly meaningful. The broader retrieval window suggests the feasibility of conducting SIF retrieval at lower resolutions, a characteristic well-suited to the ASD spectrometer. However, this same characteristic imposes challenges on FLD-based algorithms. The sFLD approach assumes constant reflectance within and outside the absorption line [23], and the 3FLD method presumes a linear relationship between reflectance and wavelength [24], failing to accommodate the substantial differences in the absorption features across this broader band. Consequently, these methods exhibit reduced fitting accuracy, with sFLD significantly overestimating SIF and 3FLD yielding suboptimal results. The complex reflectance characteristics of the “red-edge” region further exacerbate these limitations, rendering FLD-based algorithms less effective for SIF retrieval in this band.

The absorption depth of the water vapor band, which is moderate compared with the O_2_-A and O_2_-B bands, provides sufficient signal strength for fluorescence retrieval [18]. Our research results also indicate that the fluorescence retrieval potential of the water vapor band is significant. On the one hand, the retrieval accuracy of various algorithms in this band is superior to that of the O_2_-B band but inferior to the O_2_-A band. On the other hand, when using SIF to characterize the photosynthetic activity and phenological growth of vegetation [34,35,36], the retrieval results in the water vapor band show consistency with the 3FLD results in the O_2_-A band. Additionally, previous studies have shown that atmospheric radiation transfer effects on the O_2_ absorption band cannot be ignored. However, in this study, we explored the non-necessity of atmospheric correction for the water vapor absorption band by simulating irradiance at different observation heights (Table A1). Our results indicate that the impact of tower-based observation height on SIF retrieval in the water vapor band is weak, and thus atmospheric correction can be neglected. The above results, to varying extents, all suggest the potential of SIF retrieval in the water vapor band.

However, fluctuations in atmospheric water vapor concentration introduce variations in absorption depth, increasing the uncertainty of SIF retrieval [19]. Especially in our ground-based tower observations, due to the differing transmission paths of upward and downward radiation, there is a direct difference in the degree of water vapor absorption between the two, which, to some extent, exacerbates the impact of water vapor absorption effects on the SIF retrieval accuracy of algorithms. This phenomenon can be observed in our results in Figure 6. Figure 6 shows that when the water vapor column concentration changes, the accuracy of certain algorithms, such as iFLD and DOAS, varies significantly. This indicates that these algorithms exhibit high sensitivity to water vapor absorption effects, and this sensitivity also applies to the differences in water vapor absorption between upward and downward radiation. In contrast, algorithms like SFM and SVD demonstrate strong robustness to changes in water vapor column concentration, suggesting they are more suitable in such complex environments. Additionally, this study observed that changes in water vapor concentration directly affect absorption depth. As water vapor concentration increases, the absorption line deepens, which can, to some extent, improve the SIF retrieval accuracy of certain algorithms [18]. However, the irradiance reduction caused by the water vapor absorption effect also weakens the SIF signal, which, in turn, makes SIF retrieval more challenging. Our results indicate that under high water vapor concentrations, several algorithms, such as sFLD, iFLD, and DOAS, exhibit higher accuracy. However, the applicability of sFLD in the water vapor band must be excluded, as despite its accuracy improving with increasing water vapor concentration, its tendency to significantly overestimate SIF remains problematic. These variations further underscore the challenges posed by atmospheric dynamics in utilizing this spectral region for SIF retrieval.

In summary, the water vapor absorption band provides advantages related to the band position, width, and absorption depth for SIF retrieval; however, the challenges associated with complex reflectance spectral shape and the relatively large variability in atmospheric water vapor concentration compromise retrieval accuracy and stability. Our study, supported by theoretical analyses, indicates that the effectiveness of SIF retrieval in this spectral region depends heavily on atmospheric conditions, SR, and water vapor variability. Future research should prioritize refining retrieval algorithms to mitigate the impact of atmospheric water vapor fluctuations and enhance retrieval accuracy across diverse environmental conditions. While this study provides valuable insights into the strengths and limitations of SIF retrieval within the water vapor band, further validation under practical scenarios is essential to improve algorithm robustness and ensure reliable applications.

## 5. Conclusions

Current research on ground-based SIF retrieval primarily focused on the O_2_-A and O_2_-B atmospheric bands, whereas this study investigated the 719 nm water vapor absorption band. Through simulations and tower-based experiments, the performance of seven SIF retrieval algorithms under varying conditions was evaluated, including water vapor column concentrations, SRs, and SNRs.

The results demonstrated that variations in water vapor column concentrations significantly impact retrieval performance, with sFLD, iFLD, and DOAS algorithms showing notably higher sensitivity to these changes. The 3FLD, pFLD, and SVD algorithms are suitable for data with SRs finer than 1 nm. The SFM algorithm exhibits outstanding accuracy across all tested conditions, while the DOAS algorithm performs better under coarser SRs. The iFLD algorithm performs poorly in the water vapor band but provides reliable retrievals at resolutions of 0.3 nm and 0.5 nm. Additionally, the water vapor band demonstrates significant potential for SIF retrieval, with the retrieval accuracy of various algorithms in this band being superior to that in the O_2_-B band but inferior to the O_2_-A band. Furthermore, it holds considerable importance for analyzing photosynthetic activity during the vegetation phenological growth stages. Overall, conducting SIF retrieval in the water vapor band using ground-based tower platform data offers substantial research value.

Notably, these conclusions are based on specific experimental conditions, including varying water vapor concentrations, SRs, and SNR; therefore, the algorithm performance may be context-dependent, and further research is necessary to validate and optimize their applicability under different environmental conditions to ensure their reliability and robustness in practical applications.

## Figures and Tables

**Table 1:** Look-up-table for the SCOPE and MODTRAN model-simulated data.

Parameter of SCOPE	Value
Chlorophyll content (Cab) (μg/cm^2^)	20, 40, 60
Dry matter content (Cdm) (g/cm^2^)	2, 10, 20
Leaf water equivalent layer (Cw) (10^−3^ cm)	5, 10, 20
Leaf cell structure index (N)	1, 2
Leaf area index (LAI)	1, 3, 5
Leaf inclination distribution function (LIDFa and LIDFb)	Spherical (a = −0.35, b = −0.15)
SIF quantum yield efficiency (Fqe)	0.01, 0.02
Parameter of MODTRAN	Value
Atmospheric temperature profile	Midlatitude summer
Aerosol model	Rural, VIS = 23 km
Total column water vapor (g/cm^2^)	1.0, 2.0, 3.0, 4.0, 5.0
View zenith angle (degree)	0
Solar zenith angle (degree)	30
Spectral resolutions (nm)	0.3, 0.5, 1.0, 3.0

**Table 2:** Information regarding the fluorescence retrieval algorithms used in this study.

Algorithm	Equation	Reference
sFLD	$SIF=\frac{I_{out}L_{in}-I_{in}L_{out}}{I_{out}-I_{in}}$	[23]
3FLD	$SIF=\frac{{\tilde{I}}_{out}L_{in}-I_{in}{\tilde{L}}_{out}}{{\tilde{I}}_{out}-I_{in}}$	[24]
iFLD	$SIF=\frac{\alpha_{R}I_{out}L_{in}-I_{in}L_{out}}{\alpha_{R}I_{out}-\alpha_{SIF}I_{in}}$	[25]
pFLD	$Similar\;to\;iFLD,\;\;\;\ddot{R}\left(\lambda \right)=\sum_{i=1}^{n}k_{i}\Phi_{i}\left(\lambda \right)$	[26]
SFM	$L\left(\lambda \right)=\frac{R\left(\lambda \right)E\left(\lambda \right)}{\pi }+SIF\left(\lambda \right)$	[27]
SVD	$L_{sensor}=\left(\sum_{i=0}^{n_{p}}\left(a_{i}\times \lambda^{i}\right)\right)\;\times \sum_{j=1}^{n_{v}}\left(b_{j}\times v_{j}\right)+SIF\times h_{F}\times T_{↑}$	[28]
DOAS	$D=\ln\left(\frac{\pi L\left(\lambda \right)}{E\left(\lambda \right)}\right)\approx \;\sum_{i=0}^{n}p_{n}\left(\lambda \right)+\frac{h_{F}SIF}{L\left(\lambda \right)}\;$	[29]

Note. E(λ)and L(λ) are the solar irradiance and surface upwelling radiance, respectively, SIF is the chlorophyll fluorescence to be retrieved, $h_{F}$ is the fluorescence shape function. For sFLD, 3FLD, iFLD, and pFLD, $I_{in}$ and $I_{out}$ are the irradiance within and outside the absorption line, $L_{in}$ and $L_{out}$ are the radiance of the canopy within and outside the absorption line, the parameters $\alpha_{R}$ and $\alpha_{SIF}$ are the ratios of reflectance and fluorescence inside and outside the absorption line, $\ddot{R}\left(\lambda \right)$ is estimated reflectance, $k_{i}$ and $\Phi_{i}$ are the weight coefficient and the principal component vector. For SFM, $R\left(\lambda \right)$ and $SIF\left(\lambda \right)$ are mathematical functions that represent reflectance and fluorescence, respectively. For SVD, $v_{j}$ represents the high-frequency variation features derived from training, and $a_{i}$ and $b_{j}$ are fitting parameters, $T_{↑}$ is the upwelling transmittance. For DOAS, $p_{n}\left(\lambda \right)$ is the mathematical function describing the reflectance shape.

**Table 3:** Retrieval windows used by each algorithm under different SR conditions.

SRs	Retrieval Windows (nm)						
	sFLD	3FLD	iFLD	pFLD	SFM	SVD	DOAS
0.3 nm	718.5_(left)_	718.6_(left)_ 719.2_(right)_	718.5_(left)_	718.5_(left)_	[716.2, 721.6]	[713.8, 733.75]	[716.2, 721.0]
0.5 nm	718.5_(left)_	718.25_(left)_ 719.5_(right)_	718.5_(left)_	718.5_(left)_	[716.2, 721.6]	[713.8, 733.75]	[716.2, 721.0]
1.0 nm	718.5_(left)_	718.5_(left)_ 719.5_(right)_	718.5_(left)_	718.5_(left)_	[716.2, 734.0]	[716, 743.5]	[716.0, 743.5]
3.0 nm	718.0_(left)_	718.0_(left)_ 721.0_(right)_	718.0_(left)_	718.0_(left)_	[713.5, 734.5]	[659.6, 768.8]	[716.0, 743.5]

Note. The absorption line wavelengths are 718.9 nm at a 0.3 nm resolution, 718.75 nm at a 0.5 nm resolution, 719 nm at a 1.0 nm resolution, and 719.5 nm at a 3.0 nm resolution (The left and right subscripts represent the absorption lines on the left and right sides of the absorption window).

**Table 4:** RRMSE of SIF retrieval based on the QE Pro and ASD spectrometer specifications.

Instrument	RRMSE (%)						
	sFLD	3FLD	iFLD	pFLD	SFM	SVD	DOAS
**QE Pro**	49.4	5.57	9.82	5.78	2.72	5.38	12.7
**ASD**	1305.44	50.45	66.89	31.75	9.08	55.4	9.56

**Table 5:** Comparison of SIF retrieval accuracy across the O_2_-B, H_2_O, and O_2_-A bands.

**SR: 0.3 nm**		**sFLD**	**3FLD**	**iFLD**	**pFLD**	**SFM**	**SVD**	**DOAS**
R^2^	O_2_-B	0.898	0.991	0.999	0.995	0.999	0.995	0.965
	H_2_O	0.962	0.999	0.999	0.999	0.999	0.999	0.996
	O_2_-A	0.999	0.999	0.999	0.999	0.999	0.999	0.999
RRMSE (%)	O_2_-B	24.004	4.498	5.957	7.488	0.909	3.346	31.109
	H_2_O	48.846	2.236	4.116	2.323	0.348	1.749	12.173
	O_2_-A	1.568	0.444	0.486	0.550	0.685	0.662	12.028
slope	O_2_-B	0.836	1.012	1.067	1.095	1.002	0.999	0.863
	H_2_O	0.69	1.019	1.041	1.014	1.000	0.987	1.079
	O_2_-A	0.99.	1.002	1.000	1.005	1.006	0.997	0.922
**SR: 3.0 nm**		**sFLD**	**3FLD**	**iFLD**	**pFLD**	**SFM**	**SVD**	**DOAS**
R^2^	O_2_-B	0.082	0.048	0.825	0.905	0.85	0.079	0.958
	H_2_O	0.71	0.45	0.991	0.972	0.991	0.707	0.99
	O_2_-A	0.962	0.999	0.999	0.999	0.998	0.999	0.998
RRMSE (%)	O_2_-B	706.571	143.280	18.864	13.248	35.214	132.349	18.349
	H_2_O	1305.935	49.322	66.363	17.14	8.466	55.387	9.116
	O_2_-A	22.502	2.662	5.197	5.848	5.650	4.924	7.264
slope	O_2_-B	0.025	0.1	0.832	1.044	0.676	0.259	0.792
	H_2_O	0.062	0.818	2.147	1.021	1.1	0.633	0.95
	O_2_-A	0.859	0.994	1.048	1.048	1.05	1.046	0.944

**Table 6:** Analysis of SIF retrieval accuracy under different weather conditions (sunny and cloudy days).

**Sunny**	**sFLD**	**3FLD**	**iFLD**	**pFLD**	**SFM**	**SVD**	**DOAS**
R^2^	0.77	0.42	0.26	0.41	0.77	0.75	0.49
RMSE (mW/m^2^/nm/sr)	6.35	0.64	0.87	0.65	0.30	0.54	0.92
**Clody**	**sFLD**	**3FLD**	**iFLD**	**pFLD**	**SFM**	**SVD**	**DOAS**
R^2^	0.60	0.16	0.10	0.17	0.74	0.50	0.27
RMSE (mW/m^2^/nm/sr)	4.96	0.73	0.91	0.73	0.26	0.76	0.98

## Data Availability

The data can be accessed via the following DOI: 10.5281/zenodo.14619953.

## Appendix A

**Table A1:** Comparison of SIF retrieval accuracy of each algorithm at 1m and 30 m tower observation heights (SR is 0.3 nm, the sampling interval is half of the resolution, and the water vapor column concentration is 3.0 g/cm^2^).

		sFLD	3FLD	iFLD	pFLD	SFM	SVD	DOAS
RMSE (mW/m^2^/nm/sr)	1.0 m	0.558	0.023	0.129	0.027	0.001	0.022	0.149
	30.0 m	0.566	0.023	0.129	0.027	0.001	0.016	0.148
